# A 20-Year Real-World Benchmark of Metastatic Uveal Melanoma Outcomes in a Tebentafusp-Inaccessible Setting: A Comprehensive Cancer Center Study †

**DOI:** 10.3390/cancers18213512

**Published:** 2026-10-31

**Authors:** Yacob Saleh, Baker Shiha, Manar Abu Awwad, Anas Zayed, Maen Hamad, Ibrahim AlNawaiseh, Mona Mohammad, Sameer Yasir, Ramiz Abuhijlih, Wafa Asha, Imad Jaradat, Abdulla Alzibdeh, Omar Jaber, Yacoub A. Yousef

**Affiliations:** 1 Department of Internal Medicine, King Hussein Cancer Center, Amman 11181, Jordan; ma.18044@khcc.jo (M.A.A.); az.12421@khcc.jo (A.Z.); mh.17723@khcc.jo (M.H.); syaser@khcc.jo (S.Y.); 2 Faculty of Medicine, University of Jordan, Amman 11942, Jordan; bakershiha@hotmail.com; 3 Department of Surgery (Ophthalmology), King Hussein Cancer Center, Amman 11181, Jordan; ia.00146@khcc.jo (I.A.);; 4 Department of Radiation Oncology, King Hussein Cancer Center, Amman 11181, Jordan; rhijlih@khcc.jo (R.A.); wa.11007@khcc.jo (W.A.); ijaradat@khcc.jo (I.J.); aa.15218@khcc.jo (A.A.); 5 Department of Pathology, King Hussein Cancer Center, Amman 11181, Jordan; oj.12785@khcc.jo

**Keywords:** uveal melanoma, metastatic, immunotherapy, treatment, pseudoprogression, Jordan

## Abstract

Background: Uveal melanoma (UM) is a rare malignancy. Despite effective local control, 10–50% of patients develop metastatic disease, which is associated with poor prognosis. Real-world data remain lacking. This study evaluated the clinical characteristics, treatment patterns, and survival outcomes of metastatic UM in a comprehensive cancer center in Jordan. Methods: This retrospective study included adult patients with metastatic UM treated and followed at King Hussein Cancer Center in Jordan (2006–2025). Baseline characteristics and treatment patterns were collected. Descriptive and inferential statistical analyses were performed in JASP v0.95.4 and R v4.5.3. A two-sided *p* < 0.05 was considered statistically significant. Results: Among 125 patients with followed UM, 31 (24.8%) had metastatic disease and were included in the analysis. Median age at diagnosis was 51 years, 61.3% were males, 61.2% had an ECOG PS 0–1, and 51.6% had hepatic-only metastases. Median time to distant metastasis was 25 months. Median OS from initial diagnosis was 35 months, while median post-metastatic OS (PM-OS) was 10 months. First-line immunotherapy was associated with improved PM-OS compared to Dacarbazine (median, 11 vs. 5 months; *p* = 0.034; 24-month PM-OS, 44.4% vs. 0%). In exploratory analysis, patients who continued immunotherapy beyond radiologic progression, consistent with pseudoprogression, had longer PM-OS than those who discontinued therapy (median, 32 vs. 6 months; Log-rank *p* = 0.020). Conclusions: In this real-world cohort, metastatic UM was associated with poor outcomes and limited access to novel therapies including Tebentafusp. First-line immunotherapy may be associated with favorable survival outcomes, and hypothesis-generating exploratory analysis suggested potential benefit from immunotherapy continuation beyond radiologic progression.

## Simple Summary

Evidence on metastatic uveal melanoma (UM) from routine oncology practice remains scarce. We studied the clinical characteristics, treatment patterns, and survival outcomes of patients with metastatic UM treated at a comprehensive cancer center in Jordan. In this real-world cohort, metastatic UM was associated with poor outcomes, with a median post-metastatic overall survival (PM-OS) of 10 months. Notably, patients who received first-line immunotherapy achieved a 24-month PM-OS rate of 44.4%, indicating durable survival in a subset of patients. Our findings also provide hypothesis-generating evidence suggesting a potential clinical benefit from continuation of immunotherapy beyond radiologic progression, consistent with the phenomenon of pseudoprogression. Finally, the study highlights the limited availability of novel therapies for metastatic UM, including Tebentafusp, underscoring persistent disparities in access to effective treatments.

## 1. Introduction

Uveal melanoma (UM) is a rare malignancy that arises from melanocytes of the uveal tract, including the iris (3–5%), ciliary body (5–8%), and choroid (90%) [1,2]. Although it is a rare disease with an incidence of 5–6 per million in the USA, it accounts for 85% of primary ocular malignancies [3]. Despite their common origin from melanocytes, uveal and cutaneous melanomas differ in their genetic profile and clinical behavior. Mutations in GNAQ and GNA11 appear to be the major contributors to the development of UM. In contrast, mutant BRAF, mutant RAS, and mutant NF1 are the most prevalent significantly mutated genes in cutaneous melanoma [4,5]. Moreover, UM is characterized by a relatively low tumor mutational burden (TMB) [6].

Local uveal melanoma can be treated with enucleation, globe-preserving surgeries, proton beam treatment, radiotherapy, or laser therapy. The majority of cases are treated with Iodine-125 brachytherapy, with no significant mortality differences among the use of different treatment options [7]. However, up to 50% of UM patients develop metastatic disease despite effective local control, mainly involving the liver, lung, and bone. The one-year overall survival rate for metastatic disease ranges from 13% to 40%, with a median survival of 6–12 months, reflecting the aggressive nature of this disease [8,9,10].

Management strategies have primarily focused on liver-directed approaches for hepatic metastases, including surgical resection, Hepatic Intra-arterial Chemotherapy (HIAC), Transarterial Chemoembolization (TACE), and Selective Internal Radiation Therapy (SIRT); however, none of these modalities has demonstrated a clear survival benefit to date [11]. In addition, conventional cytotoxic chemotherapy agents such as Dacarbazine, Temozolomide, and Cisplatin have shown limited clinical effects with response rates ranging from only 0% to 15% [12].

Several Immune Checkpoint Inhibitors have been approved for the treatment of metastatic cutaneous melanoma, including anti-CTLA-4 monoclonal antibodies and programmed death-1 (PD-1) inhibitors. Reported objective response rates range from 10.9% to 15.2% with anti-CTLA-4 agents and from 19% to 52% with anti-PD-1 therapies, with one-year overall survival rates approaching 74.1% in patients treated with PD-1 inhibitors [13,14,15]. In contrast, no large randomized clinical trials evaluating checkpoint inhibitor efficacy have been completed in metastatic UM, and the available evidence is derived primarily from small prospective cohorts and retrospective analyses [16].

More recently, Tebentafusp, a T-cell receptor–bispecific molecule, emerged as the first-in-class agent to demonstrate a significant overall survival benefit in metastatic UM in a phase III randomized trial with clinical activity in patients expressing HLA-A*02:01, a genotype present in approximately 45% of individuals in the United States and Europe [17,18]. Unfortunately, access to Tebentafusp in Jordan is restricted primarily because of financial constraints related to HLA typing and drug procurement.

This study aims to characterize the clinical features, treatment patterns, and survival outcomes of patients with metastatic uveal melanoma treated at King Hussein Cancer Center (KHCC), the largest comprehensive cancer referral center in Jordan. To our knowledge, this is the first report from Jordan and adds to the limited real-world evidence, especially from countries where access to novel therapies, including Tebentafusp, remains not readily available.

## 2. Materials and Methods

### 2.1. Study Design

This is a single-center retrospective study that was conducted in accordance with the Declaration of Helsinki and its subsequent amendments. The study was reviewed and approved by the Institutional Review Board of KHCC, Amman, Jordan. The study identification number is 25KHCC283, approval date: 1 December 2025. Individual consent for this retrospective analysis was waived due to the retrospective nature of the study, and all data were anonymized to ensure confidentiality.

### 2.2. Participants

Adult patients with pathologically confirmed metastatic UM who received treatment at KHCC and had at least one subsequent follow-up assessment at our institution between 1 January 2006 and 31 December 2025 were included in the study. The decision to administer systemic therapy was based on the treating physician’s assessment of patient comorbidities and performance status, anticipated systemic therapy tolerability, and shared decision-making with the patient. All patients with metastatic disease diagnosed in or after 2018, when first-line immunotherapy became available at KHCC, were evaluated for treatment with immunotherapy. Patients with missing data on systemic therapy or those lost to follow-up were excluded.

### 2.3. Data Collection

Data was collected from KHCC medical records by the coauthors, and included various patients’ characteristics such as age at diagnosis, gender, ECOG performance score, metastatic staging categorized according to the size of the largest metastatic lesion (M1a, M1b, M1c) in accordance with the AJCC 8th edition, locations of metastasis, first-line systemic treatments, subsequent-line systemic treatments, and if any additional local interventions administered during metastatic-disease management. In accordance with our institutional IRB guidelines for retrospective studies, data extraction was performed using a predefined standardized data collection form to ensure consistency, all collected data were verified by the first/corresponding author; at least 20% of the collected data was cross-checked by this author to ensure accuracy and reliability, and any discrepancies were resolved by review of the original medical record and discussion between the first/corresponding author and the investigator who performed the data collection.

### 2.4. Statistical Analysis

A descriptive analysis was performed to summarize the various patients’ characteristics, including age at diagnosis, gender, ECOG performance score, metastatic staging categorized according to the size of the largest metastatic lesion (M1a, M1b, M1c), locations of metastasis, first-line systemic treatments, subsequent-line systemic treatments, and the additional metastasis-directed local interventions.

Objective response rate (ORR) was defined as the proportion of patients achieving a complete response (CR) or partial response (PR) as their best overall response according to RECIST v1.1 criteria [19]. Disease control rate (DCR) was defined as the proportion of patients achieving a CR, PR, or stable disease (SD) as their best overall response according to RECIST v1.1 criteria [19]. Comparisons of ORR and DCR between treatment groups were performed using Fisher’s exact test, as appropriate for small sample sizes.

Time to metastasis was defined as the interval between the initial diagnosis of uveal melanoma disease and the subsequent detection of metastatic disease. Overall survival (OS) was defined as the time from the initial diagnosis of UM to any-cause mortality. Post-metastasis overall survival (PM-OS) was defined as the time from the date of radiologic detection of metastatic uveal melanoma, according to RECIST v1.1 criteria, to death from any cause [19]. Survival outcomes were estimated using the Kaplan–Meier method, and survival comparisons were conducted using the Log-rank test and Cox proportional hazards regression. Observations with missing data were excluded from the Cox proportional hazards regression model.

Pseudoprogression was defined as radiologic disease progression according to RECIST v1.1 criteria, in the absence of clinical deterioration as determined by the treating physician, and subsequent continuation of immunotherapy. A time-dependent Cox proportional hazards model was performed to evaluate potential violations of the proportional hazards assumption and to characterize temporal variations in the association between pseudoprogression and survival outcomes. Observations with missing data were excluded from the time-dependent Cox proportional hazards model.

A two-sided *p*-value of <0.05 was considered statistically significant. All statistical analyses were performed in JASP version 0.95.4 and R version 4.5.3.

## 3. Results

### 3.1. Baseline Characteristics

A total of 215 patients with uveal melanoma were treated at KHCC. Among them, 86 patients with localized disease and four patients with metastatic disease were lost to follow-up. The four metastatic patients did not receive any systemic treatment and had no subsequent follow-up information available. Because treatment exposure and survival outcomes could not be reliably ascertained, these cases were excluded to avoid inaccurate survival estimates.

Consequently, 125 patients with uveal melanoma were treated and followed at KHCC. Institutional surveillance for metastatic disease consisted of routine liver ultrasonography and remained unchanged throughout the study period. Thirty-one patients (24.8%) developed metastatic disease and were included in the present study, including two patients who presented with synchronous metastatic disease at the time of uveal melanoma diagnosis (Figure 1).

The median age at diagnosis was 51 years (range: 21–92; 61.3% were males). An ECOG performance status of 0–1 was observed in 61.2% of patients, whereas 25.8% had a performance status of 2–4; ECOG performance status data were missing for 13% of patients.

Hepatic-only metastases were present in 51.6% of patients, while 38.7% had combined hepatic and extrahepatic metastases. Extrahepatic-only metastases were observed in 9.7% of patients and involved the lungs, distant lymph nodes, and peritoneum. According to metastatic staging, 54.8% of patients were classified as M1a, 29.0% as M1b, and 9.7% as M1c disease.

Importantly, first-line treatment consisted predominantly of immunotherapy, with Pembrolizumab administered to 15 patients (48.4%) and the combination of Nivolumab plus Ipilimumab administered to four patients (12.9%), resulting in a total of 19 patients receiving immunotherapy. In contrast, only three patients (9.7%) received Dacarbazine chemotherapy, while one patient (3.2%) received the combination of Darovasertib plus Crizotinib, and best supportive care alone was provided to eight patients (25.8%). The median number of cycles administered for first-line treatments was 4.5 (range: 1–35).

Only 25.7% of patients received second-line therapies that consisted of Dacarbazine chemotherapy in seven patients and Nivolumab plus Ipilimumab in one patient. Moreover, only 6.4% of all patients could reach third-line therapies, which included Carboplatin plus Paclitaxel chemotherapy in one patient and Single-agent paclitaxel in one patient.

Additional metastasis-directed local interventions were administered to selected patients and included radiotherapy in nine patients (29.0%), microwave ablation in one patient (3.2%), and surgical resection in one patient (3.2%). Among those receiving radiotherapy, treatment sites included the spine (n = 4), liver (n = 3), and brain (n = 2). Baseline patients’ characteristics are summarized in (Table 1). Additional baseline characteristics are provided in Supplementary Materials (Table S1).

### 3.2. Response to First-Line Treatments

The ORR was 11.8% in the immunotherapy group and 50.0% in the Dacarbazine group; however, this difference was not statistically significant (Fisher’s exact test, *p* = 0.298, 95%CI −6.433 to 2.751). Additionally, the DCR was 17.6% in the immunotherapy group and 50.0% in the Dacarbazine group; however, this difference was also not statistically significant (Fisher’s exact test, *p* = 0.386, 95%CI −5.967 to 3.108). Interpretation of these findings is limited by the very small number of patients treated with Dacarbazine chemotherapy.

In detail, the 19 patients who received first-line immunotherapy, two achieved a partial response, one had stable disease, one received two cycles and died before a formal response assessment could be performed, and one discontinued after a single cycle due to intolerance and transitioned to best supportive care. Fourteen patients exhibited radiological DP; however, nine of these patients had no clinical deterioration and were therefore considered to have pseudoprogression, with immunotherapy subsequently continued. Among the three patients treated with first-line Dacarbazine, one had DP, one discontinued therapy after the first cycle due to poor tolerance and transitioned to best supportive care, and one achieved a partial response. The single patient treated with first-line Darovasertib plus Crizotinib demonstrated DP on the first post-treatment CT evaluation.

### 3.3. Survival Outcomes

At a median follow-up of 87 months (range, 10–232), the median time to distant metastasis was 25 months. In the overall cohort, the median OS from initial UM diagnosis was 35 months, while median post-metastatic OS (PM-OS) was 10 months.

In univariable analyses, age was not associated with PM-OS (HR 1.001, 95%CI 0.975–1.028; *p* = 0.950), nor were sex (HR 0.92, 95%CI 0.399–2.145; *p* = 0.855), smoking history (HR 1.033, 95%CI 0.424–2.514; *p* = 0.944), metastatic stage (HR 1.939, 95%CI 0.536–7.013; *p* = 0.342), or location of metastasis (HR 1.139, 95%CI 0.251–5.171; *p* = 0.984). In contrast, ECOG performance score was significantly associated with PM-OS (HR 0.643, 95%CI 0.453–0.911; *p* = 0.009).

First-line immunotherapy was associated with a statistically significant improvement in PM-OS, with a median survival of 11 vs. 5 months in the Dacarbazine cohort (HR 0.24, 95%CI 0.061–0.991; *p* = 0.034). Consistently, the 24-month PM-OS rate was 44.4% in the immunotherapy group compared to 0% in the Dacarbazine group (Figure 2). However, in the multivariable Cox proportional hazards model adjusting for ECOG performance score, first-line immunotherapy was not independently associated with improved PM-OS compared to Dacarbazine (HR 3.82, 95%CI 0.265–55.243). The discordance between univariable and multivariable analyses, together with the markedly wide confidence intervals, likely reflects the limited sample size and the very small number of patients in the dacarbazine group (n = 3), resulting in model instability and imprecise effect estimates. In addition, the multivariable model was performed in 22 patients with 14 death events (seven events per variable), below the conventionally recommended threshold for robust Cox regression modeling. Therefore, these findings should be interpreted cautiously.

Notably, the patients who continued first-line immunotherapy despite radiological DP, consistent with pseudoprogression, demonstrated significantly improved PM-OS by log-rank analysis compared with those who discontinued immunotherapy upon radiological DP (median 32 vs. 6 months; *p* = 0.020) (Figure 3). Time-dependent Cox regression, performed in 14 patients with nine death events, demonstrated evidence of non-proportional hazards for pseudoprogression. Although the group of patients who continued first-line immunotherapy despite radiological DP, consistent with pseudoprogression, showed a trend to lower baseline hazard of death (HR 0.006, 95%CI 0.02–1.85; *p* = 0.08), the effect attenuated over time (time-dependent HR 5.263, 95%CI 0.57–50; *p* = 0.14). Given the small sample size and wide confidence intervals, these findings should be interpreted with caution and considered exploratory in nature.

## 4. Discussion

In this real-world cohort of patients with metastatic UM, the median age at diagnosis was 51 years, which is younger than that reported in North American and European cohorts [20,21]. This finding may be explained by the younger age structure of the Jordanian population and the relatively small sample size of the study. Notably, a similarly younger age at diagnosis has been reported in a Saudi Arabian cohort, suggesting that this observation may represent a broader regional pattern rather than an isolated finding [22]. Median OS from time of initial diagnosis, including the localized disease stage, was 35 months, whereas the median post-metastatic overall survival (PM-OS) was 10 months, underscoring the aggressive clinical course of the disease and aligning with previously published historical data [8,9,23]. In unadjusted analysis, first-line immunotherapy was associated with a longer median PM-OS compared with Dacarbazine (11 vs. 5 months; *p* = 0.034), and a 24-month PM-OS rate of 44.4% versus 0%, respectively. These findings suggest a potential survival benefit with first-line immunotherapy and are consistent with contemporary prospective trials and real-world series, including the GEM-1402 phase II study, which reported a median OS of 12.7 months with first-line Nivolumab plus Ipilimumab [16]. Similarly, a large Japanese meta-analysis including 41 cohorts and 1414 patients with metastatic uveal melanoma treated with immunotherapy reported a pooled median OS of 11.2 months [24]. Importantly, the comparison of first-line systemic therapies should be interpreted within the context of two distinct treatment eras at our institution, with patients in the earlier period predominantly receiving first-line Dacarbazine chemotherapy before the introduction of immunotherapy in 2018, whereas immunotherapy-based treatments were adopted thereafter into routine clinical practice. Tebentafusp, a T-cell receptor–bispecific molecule, is the first systemic therapy to demonstrate an overall survival benefit in metastatic uveal melanoma compared with investigator’s choice therapy (Pembrolizumab, Ipilimumab, or Dacarbazine). Based on the results of a phase III trial, it is approved for the treatment of previously untreated, HLA-A02:01-positive adults with metastatic UM and is recommended as the preferred first-line systemic therapy in both the ESMO–EURACAN and NCCN clinical practice guidelines [25,26,27]. Additionally, a propensity score-weighted analysis demonstrated superior overall survival with Tebentafusp compared with first-line Nivolumab plus Ipilimumab in metastatic UM, further supporting Tebentafusp as the standard first-line treatment for eligible patients [28]. Unfortunately, HLA-A02:01 typing was not performed in our cohort as Tebentafusp is unavailable at our institution, largely because of resource and financial constraints. These limitations highlight the ongoing disparities in access to novel therapies and underscore the need to improve treatment availability and enhance the inclusion of patients from resource-limited settings in future clinical trials.

Baseline ECOG performance status emerged as a strong independent prognostic factor for survival in our cohort. Although first-line immunotherapy was associated with longer PM-OS compared with Dacarbazine in unadjusted analysis, this association was not maintained after adjustment for ECOG performance status, suggesting that functional status substantially influenced observed outcomes. This likely reflects confounding by indication, whereby patients with better baseline functional status were preferentially selected for immunotherapy. This is consistent with prior metastatic UM cohorts demonstrating that performance status at the time of metastatic diagnosis independently predicts survival [29]. Together, these findings highlight the importance of patient selection in real-world treatment patterns and underscore the need for prospective studies to better define the independent survival impact of immunotherapy in this population.

A potentially novel observation in our cohort was the hypothesis-generating exploratory analysis suggestive of improved survival among patients who continued first-line immunotherapy despite radiologic progression consistent with pseudoprogression. Patients who remained on immunotherapy demonstrated longer PM-OS compared with those who discontinued treatment at the time of radiologic disease progression (median 32 vs. 6 months; Log-rank *p* = 0.020). One of the limitations of these findings is that this association was not maintained in time-dependent Cox regression modeling, likely reflecting limited sample size, potential violation of proportional hazards assumptions, and the phenomenon of immortal time bias, whereby patients who survived long enough to be observed continuing therapy necessarily had longer survival. Lead time bias may also have contributed, as patients selected to continue therapy were clinically stable and able to undergo repeat imaging. As a result, some of the apparent benefits may reflect selection of patients with inherently more indolent disease biology rather than a true treatment effect. These findings should be interpreted cautiously given the exploratory nature of the analysis and should be hypothesis-generating of a potential clinical benefit associated with immunotherapy continuation in selected patients with pseudoprogression.

With immunotherapy, a subset of patients may demonstrate radiologic enlargement of existing lesions or the appearance of new lesions that do not reflect true tumor progression, a phenomenon referred to as pseudoprogression. While pseudoprogression is well described in cutaneous melanoma, reports in UM remain limited, with only isolated case reports published to date, including a patient treated with Nivolumab in 2017 and another treated with Pembrolizumab in 2023 [30,31]. Our findings provide additional real-world evidence suggesting that pseudoprogression can occur in UM. Accurate identification of pseudoprogression remains challenging when conventional RECIST criteria are applied to patients treated with immunotherapy. Immune-adapted response frameworks, such as iRECIST, have been proposed to better distinguish atypical immune-related responses from true disease progression. In advanced melanoma, a retrospective cohort study suggested that lowering the iRECIST progression threshold and incorporating early sequential restaging scans may improve detection of pseudoprogression and are associated with more favorable survival outcomes [32]. Although these data are derived from cutaneous melanoma, they highlight the limitations of standard response criteria in the immunotherapy era and support further evaluation of immune-modified imaging approaches in UM to better characterize delayed treatment effects and refine prognostic stratification.

This study has several limitations that should be acknowledged. First, the retrospective single-center design introduces the potential for selection bias, unmeasured confounding, and limited generalizability. Second, the relatively small sample size limited statistical power and constrained the robustness of multivariable analyses, increasing the risk of model overfitting and imprecise effect estimates. Third, although ECOG performance status was incorporated into the analyses, residual confounding related to baseline functional status and treatment selection cannot be excluded, particularly given the nonrandomized nature of treatment allocation. In addition, HLA profiling was not performed, and Tebentafusp was not available at our center, limiting the applicability of our findings to contemporary biomarker-driven treatment paradigms. Finally, exploratory analyses evaluating outcomes among patients who continued immunotherapy beyond radiologic progression, consistent with pseudoprogression, may have been affected by immortal time and lead-time biases, and therefore should be interpreted as hypothesis-generating, as mentioned earlier.

## 5. Conclusions

This study provides real-world evidence on metastatic uveal melanoma patients treated at a comprehensive cancer center in Jordan, a resource-constrained country. Despite the persistently poor prognosis associated with metastatic disease and limited availability of newer therapeutic options such as Tebentafusp, our findings suggest that immunotherapy may offer improved survival outcomes when used in the first-line setting. Furthermore, exploratory analysis suggests that some patients may derive clinical benefit from continued immunotherapy despite initial radiologic disease progression, a finding that may be attributable to pseudoprogression. Although hypothesis-generating and limited by the retrospective design and limited sample size, these observations highlight the importance of careful, individualized treatment decisions in appropriately selected patients and add to the limited body of real-world evidence in this rare malignancy.

## Figures and Tables

**Figure 1:**
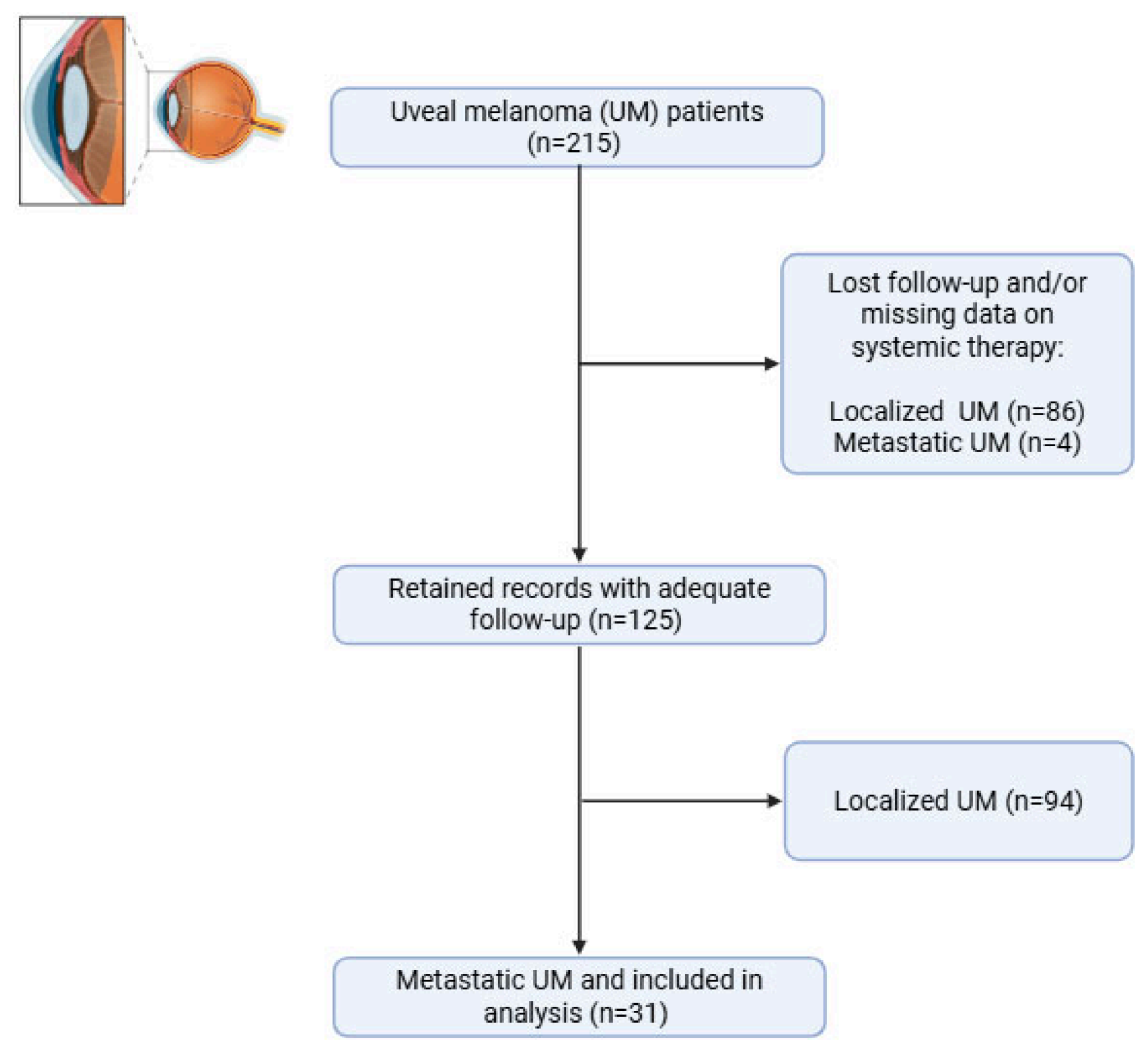
Study cohort flow diagram.

**Figure 2:**
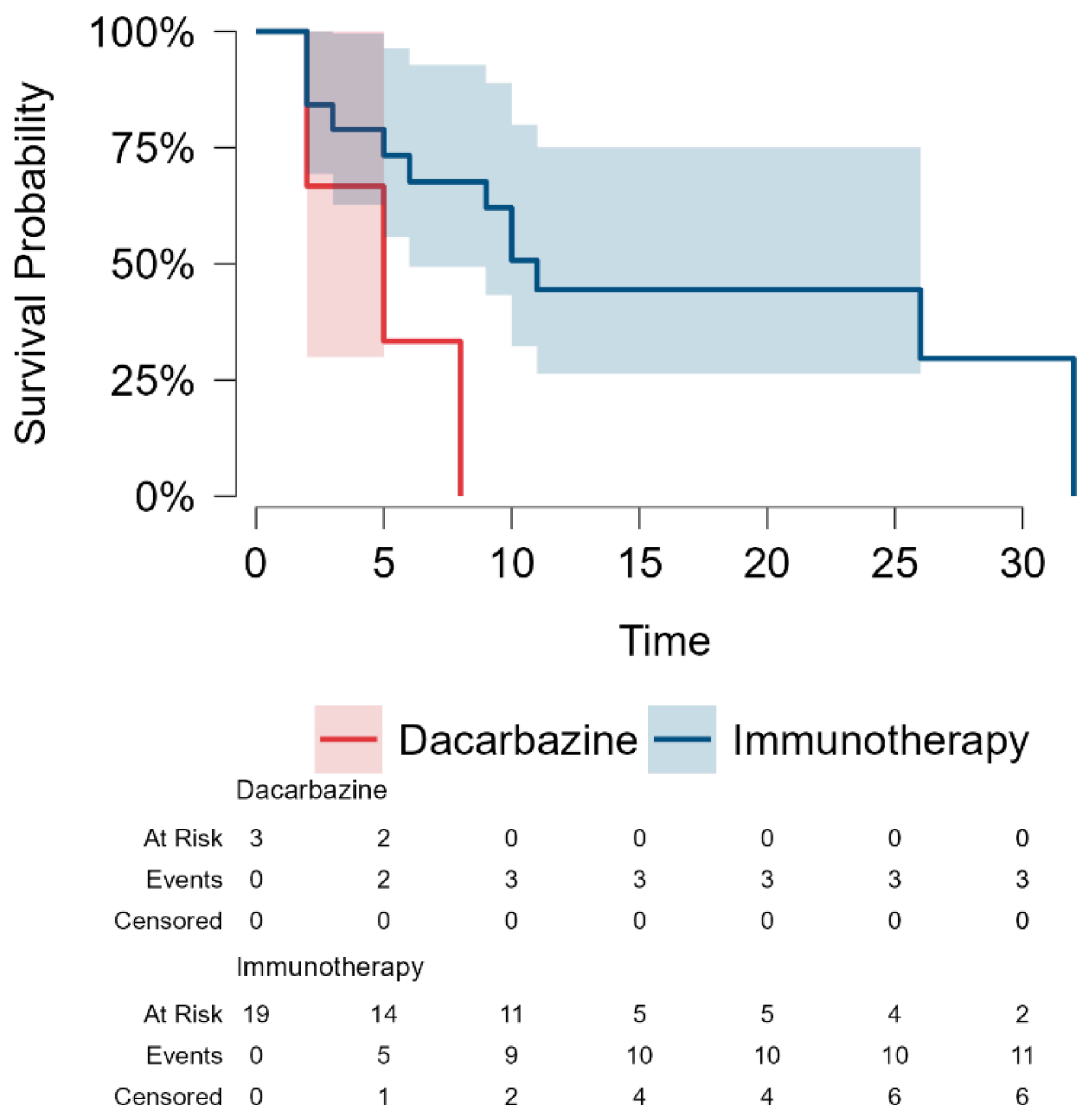
First-line treatments PM-OS.

**Figure 3:**
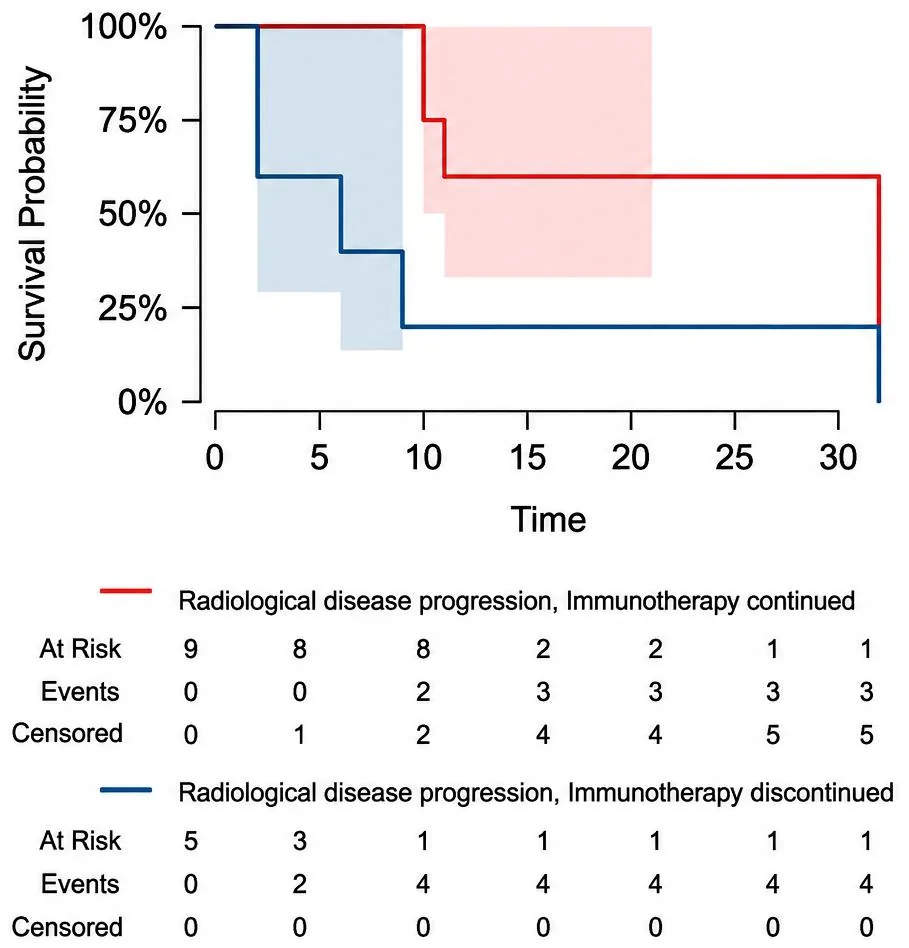
PM-OS for first-line immunotherapy following radiological disease progression.

**Table 1:** Baseline patients’ characteristics (n = 31).

Variable	Value	Total n (%)
Median age	51 years (range, 21–92).	
Sex	Male	19 (61.3%)
	Female	12 (38.7%)
Smoking history	Yes	12 (38.7%)
	No	16 (51.6%)
	missing	3 (9.7%)
ECOG performance score	0–1	19 (61.2%)
	2–4	8 (25.8%)
	missing	4 (13%)
Sites of metastasis	Hepatic-only	16 (51.6%)
	Combined Hepatic and Extrahepatic	12 (38.7%)
	Extrahepatic-only	3 (9.7%)
Stage of metastasis (AJCC 8th edition)	M1a	17 (54.8%)
	M1b	9 (29.0%)
	M1c	3 (9.7%)
	missing	2 (6.5%)
First-line treatments	Pembrolizumab	15 (48.4%)
	Nivolumab plus Ipilimumab	4 (12.9%)
	Dacarbazine chemotherapy	3 (9.7%)
	Darovasertib plus Crizotinib	1 (3.2%)
	Best supportive care alone	8 (25.8%)
Second-line treatments	Dacarbazine chemotherapy	7 (22.5%)
	Nivolumab plus Ipilimumab	1 (3.2%)
Third-line treatments	Carboplatin plus Paclitaxel	1 (3.2%)
	Single-agent paclitaxel	1 (3.2%)
Additional metastasis-directed local interventions	Radiotherapy	9 (29.0%)
	Microwave ablation	1 (3.2%)
	Surgical resection	1 (3.2%)

## Data Availability

The data presented in this study are available from the corresponding author upon reasonable request. The data are not publicly available due to institutional and ethical restrictions governing the sharing of participant data.

## Supplementary Materials

The following supporting information can be downloaded at https://www.mdpi.com/article/10.3390/cancers18213512/s1: Table S1: Baseline pathological primary tumor characteristics (N = 31).

## Abbreviations

The following abbreviations are used in this manuscript:

UM	Uveal Melanoma
PM-OS	Post Metastatic–Overall Survival
OS	Overall Survival
ECOG PS	Eastern Cooperative Oncology Group Performance Status
GNAQ	G Protein Subunit Alpha q
GNA11	G Protein Subunit Alpha 11
BRAF	B-Raf Proto-Oncogene
RAS	Rat Sarcoma
NF1	Neurofibromin 1
TMB	Tumor Mutational Burden
HIAC	Hepatic Intra-Arterial Chemotherapy
TACE	Transarterial Chemoembolization
SIRT	Selective Internal Radiation Therapy
CTLA	Cytotoxic T-Lymphocyte-Associated Protein
PD1	Programmed Cell Death Protein 1
HLA	Human Leukocyte Antigen
KHCC	King Hussein Cancer Center
IRB	Institutional Review Board
AJCC	American Joint Committee on Cancer
ORR	Objective Response Rate
CR	Complete Response
PR	Partial Response
SD	Stable Disease
DCR	Disease Control Rate
RECIST	Response Evaluation Criteria in Solid Tumors
iRECIST	Immune Response Evaluation Criteria in Solid Tumors
JASP	Jeffreys’s Amazing Statistics Program
HR	Hazard Ratio
CI	Confidence Interval
