# Supplementary material for: A 20-Year Real-World Benchmark of Metastatic Uveal Melanoma Outcomes in a Tebentafusp-Inaccessible Setting: A Comprehensive Cancer Center Study
Source: Cancers (Basel). 2026 Oct 31;18(21):3512. doi: 10.3390/cancers18213512 (PMC13761631; doi:10.3390/cancers18213512)
Supplement: Supplementary file 1 [file cancers-18-03512-s001.zip › cancers-4461756-supplementary.pdf]

Supplementary Table S1. Baseline pathological primary tumors characteristics (N=31)

Variable	Value	Total n(%)
Primary tumor site	Choroidal	18 (58.1%)
	Ciliochorodial	6 (19.4%)
	Ciliary body	2 (6.5%)
	missing	5 (16.0%)
Median tumor thickness (mm)	10.35 (range, 2.6-16)	
Median largest tumor base diameter (mm)	17.4 (range, 9.6-23)	
Histological subtype	Epithelioid	4 (12.9%)
	Spindle cell	4 (12.9%)
	Mixed cell type	4 (12.9%)
	missing	19 (61.3%)
Mitotic rate (/40 HPFs)	4.5 (range, 1-25)	
Pathological T stage (AJCC 8th edition)	T3	2 (6.5%)
	T4	10 (32.2%)
	missing	19 (61.3%)
Extrascleral extension	Yes	3 (9.7%)
	No	9 (29%)
	missing	19 (61.3%)
